# Review of low-cost light sources and miniaturized designs in photoacoustic microscopy

**DOI:** 10.1117/1.JBO.29.S1.S11503

**Published:** 2023-10-20

**Authors:** Bingxin Huang, Terence T. W. Wong

**Affiliations:** a Hong Kong University of Science and Technology, Department of Chemical and Biological Engineering, Translational and Advanced Bioimaging Laboratory, Hong Kong, China; b Hong Kong University of Science and Technology, Research Center for Medical Imaging and Analysis, Hong Kong, China

**Keywords:** photoacoustic microscopy, low-cost light sources, miniaturized designs, microvasculature imaging

## Abstract

**Significance:**

Photoacoustic microscopy (PAM) is a promising imaging technique to provide structural, functional, and molecular information for preclinical and clinical studies. However, expensive and bulky lasers and motorized stages have limited the broad applications of conventional PAM systems. A recent trend is to use low-cost light sources and miniaturized designs to develop a compact PAM system and expand its applications from benchtop to bedside.

**Aim:**

We provide (1) an overview of PAM systems and their limitations, (2) a comprehensive review of PAM systems with low-cost light sources and their applications, (3) a comprehensive review of PAM systems with miniaturized and handheld scanning designs, and (4) perspective applications and a summary of the cost-effective and miniaturized PAM systems.

**Approach:**

Papers published before July 2023 in the area of using low-cost light sources and miniaturized designs in PAM were reviewed. They were categorized into two main parts: (1) low-cost light sources and (2) miniaturized or handheld designs. The first part was classified into two subtypes: pulsed laser diode and continuous-wave laser diode. The second part was also classified into two subtypes: galvanometer scanner and micro-electro-mechanical system scanner.

**Results:**

Significant progress has been made in the development of PAM systems based on low-cost and compact light sources as well as miniaturized and handheld designs.

**Conclusions:**

The review highlights the potential of these advancements to revolutionize PAM technology, making it more accessible and practical for various applications in preclinical studies, clinical practice, and long-term monitoring.

## 1 Introduction

Photoacoustic tomography (PAT), as a rapidly growing biomedical imaging technique, provides structural, functional, hemodynamic, molecular mechanism information.^1^^–^^3^ In PAT, when a pulsed or modulated light beam is irradiated on the tissue, endogenous or exogenous absorbers in the tissue absorb light and convert the photon energy into heat, resulting in a rapid temperature rise. The subsequent thermoelastic expansion of the tissue induces the generation of acoustic waves, which are also termed PA waves.^4^ Therefore, PAT combines the advantages of both wavelength-specific optical absorption contrast and ultrasound deep imaging depth, which has made it a promising modality in cardiology, oncology, and other applications from bench to bedside over the past few decades.^5^^–^^10^

There are two major PAT system implementations: photoacoustic computed tomography (PACT) and photoacoustic microscopy (PAM). In PACT systems, high-power lasers are used to cover the entire region of interest and induce the PA waves. Typically, a multi-element (linear array,^11^ arc array,^12^ or hemisphere array^13^) ultrasonic transducer (UT) is used to collect the PA waves in parallel for tomographic or cross-sectional image reconstruction. PACT systems typically use low-frequency transducers to achieve deep-tissue imaging. Unlike PACT, PAM systems usually utilize confocal geometry (i.e., co-axially focus the excitation light beam and acoustic detection), resulting in high spatial resolution and signal-to-noise ratio (SNR). The lateral resolution of PAM systems is dependent on either the focusing of the excitation light beam or the acoustic detection, referring to optical-resolution PAM (OR-PAM) and acoustic-resolution PAM, respectively. PAM has been widely applied to imaging fine structures both *ex vivo* and *in vivo* due to its high resolution and rich optical contrast.^14^^–^^20^ Meanwhile, PAM has undergone tremendous technological advancements in improving spatial resolution,^21^^,^^22^ imaging speed,^17^^,^^20^^,^^23^^,^^24^ penetration depth,^25^ and contrast.^18^ However, most PAM systems are limited to bench forms due to the requirements of bulky and expensive lasers, bulky motorized stages for two-dimensional (2D) or three-dimensional (3D) scanning, and complex designs for combining the light beam and the ultrasound in confocal geometry. To circumvent these requirements and expand the applications of PAM, various research directions have been proposed to implement low-cost, compact, and fast PAM imaging devices.

Here, we review the recent advances in the development and applications of PAM systems with inexpensive components and small sizes. In this review, we first describe the principle and main components of PAM and discuss the limitations of conventional PAM systems in Sec. 2; Sec. 3 is dedicated to PAM systems with low-cost and compact laser sources; Sec. 4 is dedicated to PAM systems with miniaturized or handheld designs; in Sec. 5, we discuss the perspective applications of these cost-efficient or miniaturized PAM systems; and finally, we conclude the review and discuss the potential challenges of these PAM systems in Sec. 6.

## 2 Principles and Main Components of PAM

PAM is based on the PA effect, which shows multiple advantages over some conventional optical imaging techniques, such as optical coherence tomography (OCT) and confocal microscopy. (1) Deep imaging depth—the detected signal is ultrasound, which is less scattered in biological tissue than light; (2) high endogenous molecular contrast—the contrast comes from the optical absorption, which has high specificity; (3) multilayer imaging with depth-resolved acoustic detection—PAM can achieve high-speed 3D imaging with 2D scanning. The PA signal amplitude (P) is expressed as^26^

$$P=\Gamma \eta_{\mathrm{th}}\mu_{a}F,$$

where Γ is the Grueneisen parameter, which is proportional to the local temperature; $\eta_{\mathrm{th}}$ is the heat conversion efficiency, which represents the portion of absorbed light energy converted into heat; $\mu_{a}$ is the optical absorption coefficient (${\mathrm{cm}}^{-1}$) of the excited biomolecules; and F is the optical fluence ($J/{\mathrm{cm}}^{-1}$).

The main components of the PAM system are excitation sources, light delivery, scanning protocol, and acoustic detection. As these components continue to advance, the PAM system has the potential to become one of the best tools for both preclinical and clinical studies. Among all of the improvements, excitation sources, light delivery, and scanning protocol are the main aspects of the strategies to optimize PAM systems, making them cost-efficient and compact. The excitation sources for PAM are usually pulsed lasers, such as Q-switched diode-pumped solid-state lasers, Ti:sapphire lasers, and optical parametric oscillators. These lasers can generate high-energy light pulses with a short pulse width to obtain PA signals with a high SNR. However, the high cost, bulky size, and high-level laser-safe requirement of these lasers prevent their wide usage in clinical environments. Recently, laser diodes (LDs) have become promising alternative laser sources for PAM systems due to their economical price and compact size, promoting the development of PAM into more cost-effective and miniaturized forms. Light delivery and the associated scanning system are used to regulate the transportation of light from the excitation light source to a target. Traditional PAM systems usually use free-space optics to deliver light and bulky motorized stages to realize 2D scanning. In recent years, the development of fiber-based optics and novel beam scanning devices has promoted the strategy for PAM miniaturization.

## 3 PAM Systems with Low-Cost and Compact Excitation Sources

To implement cost-effective and compact PAM systems, LDs can be an alternative excitation source due to their low cost, small size, high repetition rate, and wide wavelength range availability. LD is a semiconductor laser device that converts the input electric energy into light energy. Allen et al.^27^ reported a preliminary study to show the potential of LDs as excitation light sources for PA signal generation. However, the low peak output power in LDs, which is to avoid catastrophic optical damage effects, is a key challenge of this technology; with LDs, thousands of times averaging PA signals over the same region are required for an acceptable SNR.^28^^,^^29^ Therefore, the development of LD-based PAM was relatively slow at the beginning. Afterward, with the rapid development of optics and electronics, many studies have focused on using LDs as a substitute for conventional lasers in PAM, promoting their clinical applications and even in low-resource settings. Both pulsed LDs (PLDs) and continuous-wave LDs (CWLDs) have been developed for PAM biomedical applications.

### 3.1 PLD-based PAM

**Fig. 1:**
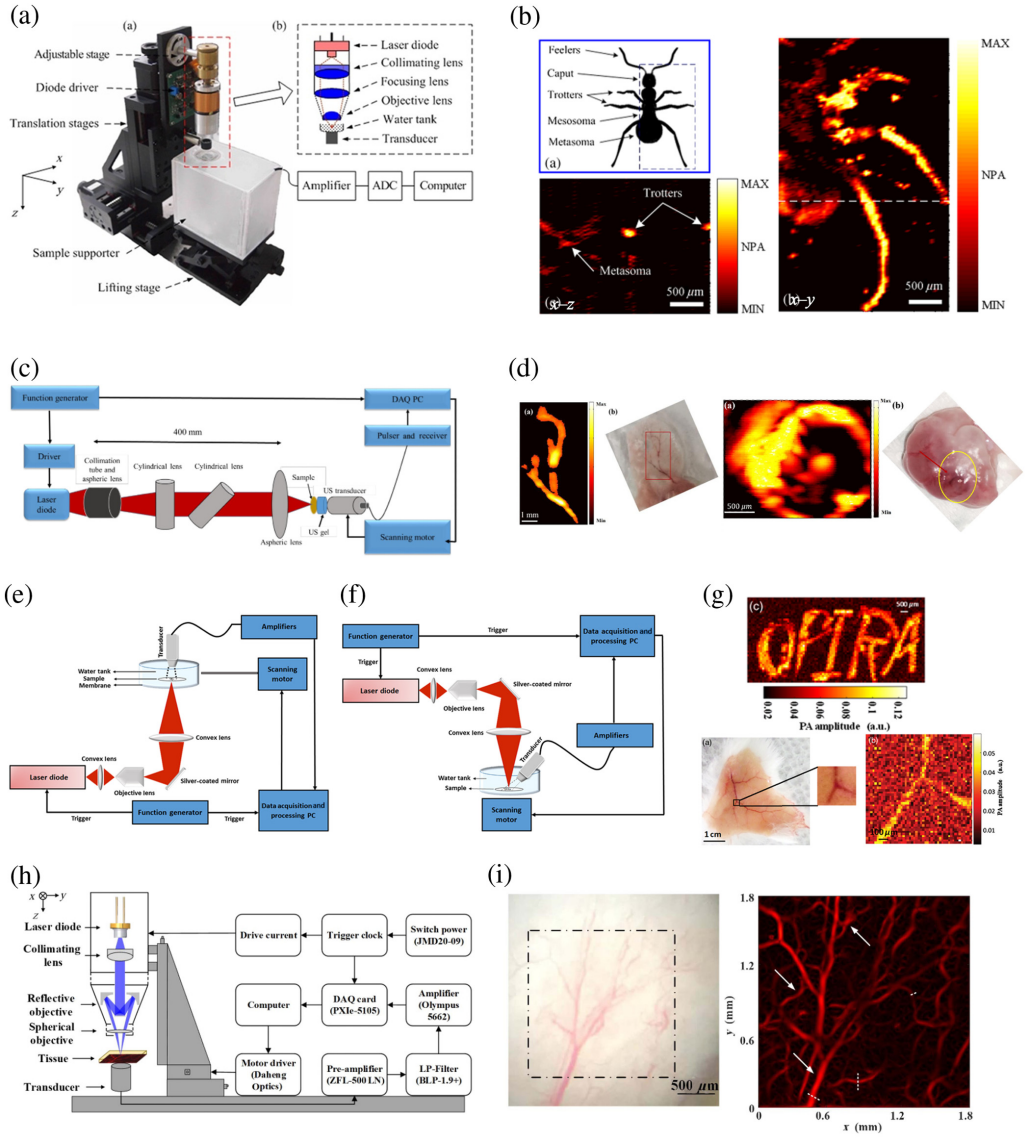
PLD-based PAM. (a) A schematic of a 905 nm PLD-based OR-PAM system. ADC, analog-to-digital converter. (b) Anatomic schematic of an ant, 2D PA MAP image of the ant, and 2D PA B-scan image along the white dashed line, respectively. NPA, normalized photoacoustic amplitude. (c) A schematic of PLD-based PAM using aspheric lens and CL for light beam collimating and focusing. DAQ, data acquisition. (d) PAM images and photographs of a mouse ear and porcine ovary, respectively. (e) A schematic of transmission-mode PLD-based PAM system. (f) A schematic of reflection-mode PLD-based PAM system. (g) Reflection-mode PLD-based PAM images of a phantom and *ex vivo* mouse skin. (h) A schematic of the VIS PLD-based PAM system using a reflective objective. (i) Photograph and VIS PLD-based PAM image of the microvascular structure in a mouse ear *in vivo*. Reproduced with permission from Refs. 32–36.

In 2012, the first attempt to visualize the blood vessel phantom in 3D was performed using a PLD at 905 nm with light focused by collimating and focusing lenses.^30^ They generated preliminary 3D volumetric renderings of the knotted and helical blood vessel phantoms, which were accurately represented compared to photographs. However, the lateral resolution of their PLD-based PAM system was 500  μm, which was not ideal for real human blood vessel imaging because some blood vessels, such as arterioles, venules, and capillaries, are extremely small, ranging from 0.5 to 30  μm in diameter.^31^ The next year, Zeng et al.^32^^,^^33^ developed a 905 nm PLD-based OR-PAM system with a 0.8 kHz repetition rate, 100 ns pulse width, and 4.9  μJ pulse energy, shown in Fig. 1(a). Compared with previous studies, they improved the lateral resolution from hundreds of microns to 1.5  μm, and the whole system was assembled in portable equipment. A dead ant (a length of ∼3.5  mm and a diameter of 0.3 to 1 mm) was imaged to demonstrate its feasibility for imaging small animals. 2D PAM images of the dead ant are shown in Fig. 1(b), which shows the ant’s size and shape. Another PLD-based PAM system, with a 1 kHz repetition rate, 124 ns pulse width, and 3  μJ pulse energy, imaged the phantoms made of polyethylene tubes filled with blood and a mouse ear to demonstrate the feasibility of imaging biological tissues.^37^ Each A-line signal was averaged 128 times to achieve ∼13  dB of SNR, and more averages are needed to further improve the SNR.

Erfanzadeh et al.^34^ came up with an optical scheme combining an aspheric lens and a cylindrical lens (CL) to efficiently collimate and focus the excitation light beam of a 905 nm PLD. As shown in Fig. 1(c), the light was first collimated by an aspheric lens and then by two perpendicularly arranged CLs in perpendicular directions. After collimation, the rectangular light beam (∼15  mm×10  mm) was focused on the sample by an aspheric lens with a numerical aperture (NA) of 0.71. A 3.5 MHz UT was used to detect the PA signal with 128 times averaging. Benefiting from the optical design, the SNR of phantom and mouse ear images was improved by at least 12 dB in comparison with the work in Ref. 37 with a similar PLD. Images of the vasculature on *ex vivo* mouse ear and porcine ovarian tissue [Fig. 1(d)] with 22 dB and 25 dB SNR, respectively, demonstrate the feasibility of this low-cost OR-PAM system for imaging biological samples and potentially characterizing ovarian cancer. However, it is hard to visualize microvasculature due to the limited resolution, showing some discontinuous or mixed blood vessels in PAM images.

Another group presented PLD-based OR-PAM systems in both transmission and reflection modes.^35^ According to the schematic of the two systems [Figs. 1(e) and 1(f)], the only difference is that the transducer was held at a 180 deg angle with the incident light in transmission mode and at a 40 deg angle in reflection mode. PAM images of tissue-mimicking phantoms and *ex vivo* mouse skin vasculature are shown in Fig. 1(g). They demonstrate the feasibility of the OR-PAM system with a very low-energy PLD (with an output peak power of ∼6  W) for imaging biological tissues. However, due to the low excitation energy, averaging numerous PA signals at each position was still performed to increase the SNR. Otherwise, complex signal processing algorithms are required to reduce the number of averaging.^38^

Motor scanning was used in the aforementioned systems, and multiple averaging was required in data acquisition (DAQ). Erfanzadeh et al.^39^ demonstrated a laser scanning LD-PAM system without signal averaging during DAQ. The 905 nm PLD beam, with a 1 kHz repetition rate and 50 ns pulse width, was well collimated and focused into a small enough beam spot for 2D galvanometer scanning. A 3.5 MHz UT was used to detect PA signals without averaging, achieving ∼370 A-lines per second. Images of the vasculature on *ex vivo* mouse ear and porcine ovarian tissue with ∼12 and ∼18  dB SNR, respectively, demonstrate the feasibility of this low-cost and fast laser scanning OR-PAM system for imaging biological samples and characterization of ovarian cancer.

Very recently, a new OR-PAM system was developed with the PLD beam homogenized and shaped by a square-core multimode optical fiber (MMF).^40^ In the PLD beam transport optics, a set of plano-convex aspheric lenses were first used for fiber coupling, maximizing the beam power to the MMF. A 20  μm circular pinhole was attached to the fiber end, followed by another set of aspheric lenses for light delivery to the samples. A 128-element linear phased array was used for the detection of PA signals. The relatively high-quality PA images of *ex vivo* rabbit ears, obtained with this fiber-coupled LD reflection-mode OR-PAM system, have shown the system’s great potential for the characterization of blood vessels and hair follicles.

The absorption coefficient of hemoglobin is much lower in the near-infrared (NIR) range than in the visible range, resulting in low image quality for blood capillary imaging when using NIR PLDs-based PAM systems. Zeng et al.^41^ developed a visible PLD OR-PAM (VIS PLD-OR-PAM) system, achieving a lateral resolution of ∼0.95  μm. The PLD was operated at a 405 nm wavelength with a 1 kHz repetition rate, 174 ns pulse width, and ∼52  nJ pulse energy (∼3 orders of magnitude less than that of NIR PLDs). Subcutaneous microvasculature on a mouse back was clearly visualized, validating the label-free imaging feasibility of the VIS PLD-OR-PAM in superficial tissues. To ensure sufficient energy density on the samples and generate effective PA signals, a PLD with low peak power necessitates a high NA objective to focus the laser beam tightly while limiting the working distance. To overcome this dilemma, Deng et al.^36^ developed a VIS PLD-OR-PAM system with a reflective objective (NA=0.3) to maximize the working distance up to 22 mm [Fig. 1(h)]. *In vivo* imaging of blood vessels and capillaries in mouse ears using this system demonstrate its potential for preclinical applications, shown in Fig. 1(i).

It is worth noting that, with these PLDs, PAM systems could be low-cost and compact due to their economical price and small volume. However, most of the commercially available PLDs are at NIR wavelengths and provide low pulse energy of several μJ, resulting in a low SNR for biological imaging. To solve this issue, Manwar et al.^42^ developed an adaptive denoising filtering method to enhance the SNR of PA signals obtained using low-energy PLDs.

### 3.2 CWLD-based PAM

By contrast, CWLDs are available in a wide range of wavelengths (visible and NIR). Visible CWLD-based PAM systems can obtain better SNR images even when the pulse energy is low due to the high absorption of blood in the visible range. Taking advantage of CWLDs, including the low cost and wide range of available wavelengths, they have been heavily explored to be used as an excitation source in PAM.

**Fig. 2:**
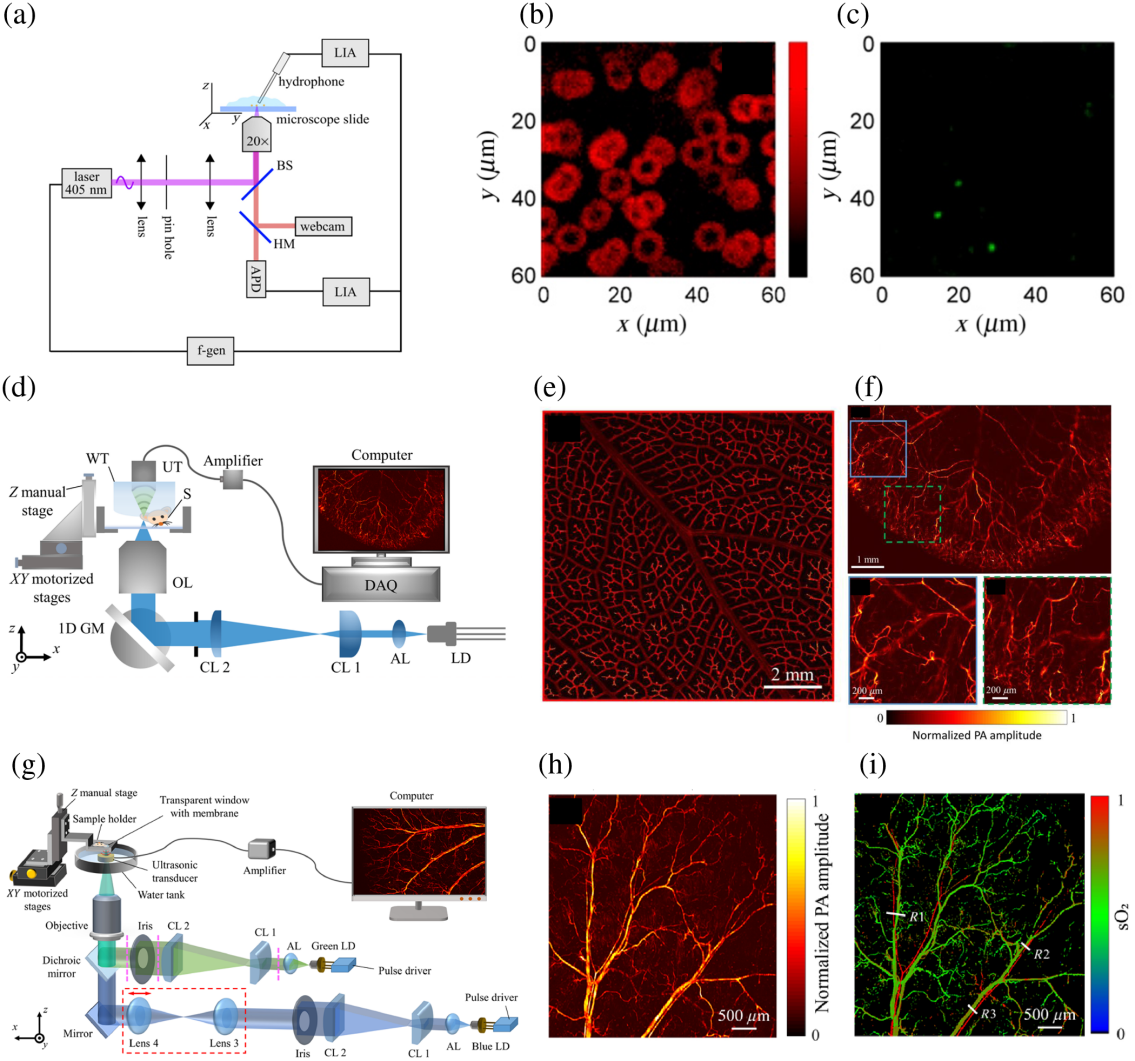
CWLD-based PAM. (a) A schematic of a 405 nm CWLD-based OR-PAM system. APD, avalanche photodiode; LIA, lock-in amplifiers; f-gen, function generator. (b) PAM image of human red blood cells. (c) Corresponding luminescence image. (d) A schematic of the high-speed high-resolution LD-based PAM system. AL, aspheric lens; CL, cylindrical lens; GM, galvanometer mirror; OL, objective lens; S, sample; WT, water tank; UT, ultrasonic transducer. (e) PAM image of a leaf phantom dyed with black ink. (f) *In vivo* PAM images of a mouse ear. (g) A schematic of the dual-wavelength LD-based PAM system. (h) *In vivo* PAM image of a mouse ear with blue LD excitation. (i) ${\mathrm{sO}}_{2}$ image of the mouse ear. Reproduced with permission from Refs. 43–45.

CWLDs are commonly operated in CW mode across various applications. CWLDs with a specific light intensity modulation scheme can be employed to generate PA signals in the frequency domain. Langer et al.^43^ incorporated a CWLD with a wavelength of 405 nm and an output power of 120 mW in a PAM system. The CWLD was modulated with a frequency of 10 MHz to facilitate PA imaging. Figure 2(a) shows the PAM image in which the distinct donut shape of red blood cells is readily discernible. In addition, another fluorescence imaging modality was also combined in their system, providing luminescence images [Fig. 2(b)] with complementary information.

Recently, the feasibility of using CWLDs in pulsed mode has been demonstrated by overdriving the diode while avoiding catastrophic optical damage and thermal damage. This approach represents a complementary solution to PLDs. Stylogiannis et al.^46^ overdrove CWLDs at 6 different wavelengths, ranging from 445 to 830 nm with a peak current up to 45 times higher than the manufacturer-specified maximum current, resulting in short pulses of about 10 ns with a pulse energy of about 200 nJ and a repetition rate of higher than 600 kHz. The diode characterization experiment shows that CWLDs, operated in pulsed mode, can provide higher optical power than those operated in CW mode, with a stable performance at different repetition rates. Combined with a raster-scanning system, the pulse-driven 450 nm CWLD was used for PA measurement to characterize their imaging performance in a reflection-mode PAM system. The lateral and axial resolutions were 110 and 33  μm, respectively. The vasculature of the mouse ear and human forearm at the epidermal and dermal layers was imaged by the CWLD-based PAM system with 500 times averaging. The SNR was observed to be 15 to 20 dB for the initial 100  μm depth and declined sharply with increasing depth, indicating the high absorption and scattering of blue light by the tissue. Another implementation of PAM, utilizing a compact fingertip 450 nm CWLD, has demonstrated the capability of both pulsed modulation and continuous modulation for PAM.^47^ However, it also suffered from suboptimal resolution and imaging speed, necessitating 256 times averaging to achieve acceptable results. To generate high-resolution PAM images with high imaging speed simultaneously, Li et al.^44^ developed a high-speed high-resolution CWLD-based PAM system for *in vivo* microvasculature imaging [Fig. 2(d)]. Considering the emitter size of LDs, they optimized the optical system by reshaping the laser beams from LDs horizontally and vertically. A pair of CLs combined with an iris was used to control the laser beam to an ideal circular shape, achieving a high lateral resolution of 4.8  μm. The maximum SNR can reach up to ∼42  dB with a pulse energy of ∼30  nJ for leaf phantom imaging [Fig. 2(e)] and ∼31  dB with a pulse energy of ∼60  nJ for *in vivo* microvasculature imaging of a mouse ear [Fig. 2(f)]. The exceptional SNR of the CWLD-based PAM system renders signal averaging unnecessary. In addition, the incorporation of a 1D galvanometer mirror scanning system enables high-speed imaging capabilities. However, the limited working distance of the high NA objective lens restricts the implementation of this system solely to transmission-mode imaging, making it unsuitable for visualizing microvasculature in thick tissues. In addition, using a single wavelength LD may preclude the estimation of crucial diagnostic parameters [such as oxygen saturation (${\mathrm{sO}}_{2}$)], which limits clinical applications of the PAM system. Therefore, they developed a reflection-mode high-resolution OR-PAM system for *in vivo* ${\mathrm{sO}}_{2}$ imaging using 2 LDs with different wavelengths, achieving a high lateral resolution of ∼6  μm, shown in Fig. 2(g).^45^ High-quality microvasculature imaging [Fig. 2(h)] and accurate ${\mathrm{sO}}_{2}$ calculation [Fig. 2(i)] can be achieved with this low-cost PAM system, which facilitates the widespread adoption of PAM in both preclinical and clinical applications.

As such, the use of CWLDs in pulsed mode has the potential to expand the range of applications for LD-based imaging techniques, particularly in the field of medical diagnostics.

With the full utilization of the Gruneisen relaxation effect, it is possible to induce the nonlinear PA effect. In this situation, the amplitude of PA signals exhibits variability depending on the baseline temperature of the tissue. Therefore, Zhong et al.^48^^,^^49^ introduced an LD-based PAM system that combines a single-wavelength PLD with several CWLDs with different wavelengths, achieving multi-wavelength PAM. The CWLDs were utilized to heat the sample, and the PA signals were generated by the single-wavelength PLD. By controlling the laser irradiation sequence (pulse-CW-pulse), PAM images before and after thermal heating can be obtained. Proper differentiation operations can be applied to reveal images with different wavelengths and known light absorption. *Ex vivo* imaging and clear identification of three tubes filled with different color ink demonstrated its capability for multi-wavelength PA imaging.

## 4 PAM Systems with Miniaturized and Handheld Designs

In addition to excitation sources, the light delivery and scanning protocol are also keys to the strategy for the miniaturization and commercialization of PAM systems. Traditional bench-top PAM systems are often bulky and complex, due to the free-space optics for light delivery and cumbersome motorized stages for scanning, which limits their portability and versatility. In recent years, there has been a growing interest in developing miniaturized PAM systems combined with fiber-based optics and novel scanning devices to maximize the potential of PAM in the biomedical imaging field. Novel scanning devices [e.g., galvanometer scanner (GVS) and micro-electro-mechanical system (MEMS) scanner] have enabled fast scanning with the miniaturized probe. In this section, we discuss recent advances in miniaturized PAM systems, focusing on light delivery and scanning protocol designs, as well as their technical challenges and biomedical applications.

### 4.1 PAM Systems with GVS

**Fig. 3:**
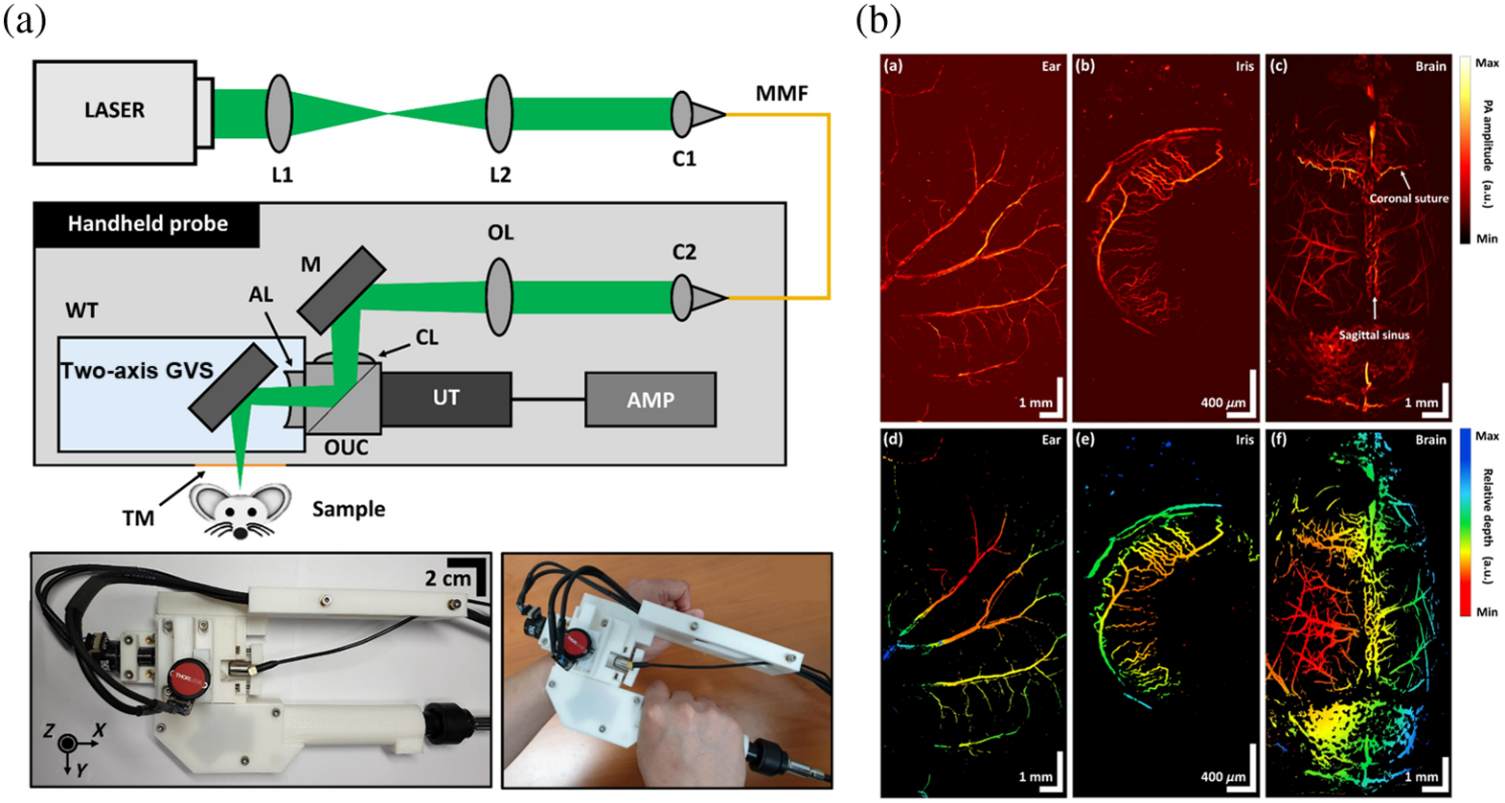
GVS-based PAM. (a) Schematic representation and photographs of the WF-GVS-HH-PAM system. AMP, amplifier; C, collimator; MMF, multi-mode fiber; OUC, opto-acoustic combiner; TM, transparent membrane; M, mirror; AL, acoustic lens; CL, correction lens; L, lens. (b) *In vivo* PAM MAP (top row) and depth-encoded (bottom row) images of microvasculature in mouse ear, iris, and brain, respectively. Reproduced with permission from Ref. 53.

In conventional PAM, 1D depth-resolved PA signals are first detected by the UT, and then two-axis motorized scanning stages are needed to obtain cross-sectional or volumetric images by linear or raster scanning. To increase the scanning speed and reduce the size of the PAM probe, GVS based on a raster scanning mechanism has been developed as a representative imaging scanner for miniaturized and handheld PAM. Hajireza et al.^50^ demonstrated a handheld real-time OR-PAM system, which unveiled a new generation of PAM in various clinical applications. A 2D GVS mirror system was used to pass and scan the 532 nm laser beam with high and low scanning rates of 400 and 1 Hz in 2 directions, respectively. Then, the laser beam passed through the image guide fiber into the handheld probe with only a 40  mm×60  mm footprint and <500  g in weight. Furthermore, this group attempted to further miniaturize the PAM probe by incorporating a graded-index lens, enabling precise focusing in a compact form.^51^ Zhang et al.^52^ also employed a field programmable gate array-driven fast 2D GVS to a compact PAM probe to facilitate high-resolution imaging of subcutaneous microvessels with deep penetration depth. It was able to capture a maximum amplitude projection (MAP) image of 400×400  pixels within a 2  mm×2  mm imaging area, with an acquisition time of 16 s. *In vivo* animal experiments and human skin imaging demonstrated the capabilities of the miniaturized PAM for microvasculature visualization as well as quantitative analysis of the diameters and depths of the blood vessels. Instead of optical scanning only, Seong et al.^53^ developed a waterproof, two-axis GVS-based handheld PAM (WF-GVS-HH-PAM) system that scans both optical and acoustic beams, providing an extended field of view (FOV) of 14.5  mm×9  mm, as shown in Fig. 3(a). By positioning the GVS at the distal end of the probe, the proposed system successfully addressed the FOV limitation encountered in previous GVS-based handheld PAM systems. The microvasculature of the mouse ear, iris, and brain were imaged by the WF-GVS-HH-PAM system with lateral and axial resolutions of 11.5 and 31.3  μm, respectively. The *in vivo* experiment results [Fig. 3(b)] demonstrate the ability of the WF-GVS-HH-PAM to visualize arteries, veins, single capillaries, and some distinctive blood vascular features (e.g., coronal suture and sagittal sinus). Furthermore, it provides the 3D volumetric and quantitative information in the depth-encoded PAM images. In 2022, Chen et al.^54^ developed a freehand scanning handheld PAM (FS-PAM) probe to achieve fast speed, great flexibility, and a large FOV in one system. Dual-wavelength (532 and 558 nm) pulsed laser beams were connected to the FS-PAM probe, providing 3D images of vasculatures with not only anatomical but also functional information (e.g., ${\mathrm{sO}}_{2}$). The FS-PAM system incorporated a hybrid resonant-GVS, enabling video camera mode imaging and facilitating simultaneous localization and mapping in PAM.

Instead of raster scanning, PAM based on a rotatory scanning mechanism has also been developed recently. A cylindrically focused transducer is used to form an acoustic focal line, which is adjusted to be confocal with the optical imaging plane. The 2D GVS scans the focused laser spot in line from the left edge to the right edge of the acoustic focal line to generate a B-scan image. Following each optical line scan, both the optical scan line and acoustic focal line rotate simultaneously by a predetermined angle. Multiple scans and rotations with a total scanning angle of 180 deg are executed to encompass the entire imaging area. Based on the rotatory scanning mechanism, Jin et al.^55^^,^^56^ reported a new portable OR-PAM (p-ORPAM). A 532 nm pulsed laser beam was coupled into a single mode fiber (SMF) and delivered into the imaging part. A 2D GVS was combined with a motorized rotator to achieve large FOV imaging. The performance of the system was evaluated in mouse ears and brains, rabbit ears and eyes, and human oral vasculature with a large FOV of up to 8.4 mm in diameter and a depth of field of 1.5 mm. Representative results demonstrate that the p-ORPAM is widely applicable to multiscale organisms. Subsequently, Qin et al.^57^ conducted a study on the structural and functional cerebral vasculature of rhesus monkeys using the improved p-ORPAM system. This research holds great significance in the field of neuroscience due to the high resemblance of rhesus monkeys’ brains to that of humans. Furthermore, Qin et al.^58^ developed a dual-modality system combining OR-PAM and spectral-domain optical coherence tomography for oral inspection, providing microvasculature and microstructure information of oral tissue simultaneously.

### 4.2 PAM Systems with MEMS Scanner

In addition to GVS, the MEMS scanner has emerged as another prominent imaging scanner for miniaturized or handheld PAM due to its metrics: (1) the MEMS scanner is compact and lightweight, making it ideal to be integrated into various devices for portable applications; (2) the MEMS scanner can be designed to various scanning modes (e.g., linear, raster, and specific patterns), showing great flexibility in imaging applications; (3) the MEMS scanner provides a high scanning speed, which holds great significance in real-time and dynamic imaging of biological structures while reducing minimal motion artifacts; and (4) the MEMS scanner requires relatively low driven voltage and consumes lower power compared with other scanning devices, which enhances the overall energy efficiency, making it suitable for handheld PAM systems. Therefore, the MEMS scanner has been widely applied in the development of miniaturized and handheld PAM systems.

**Fig. 4:**
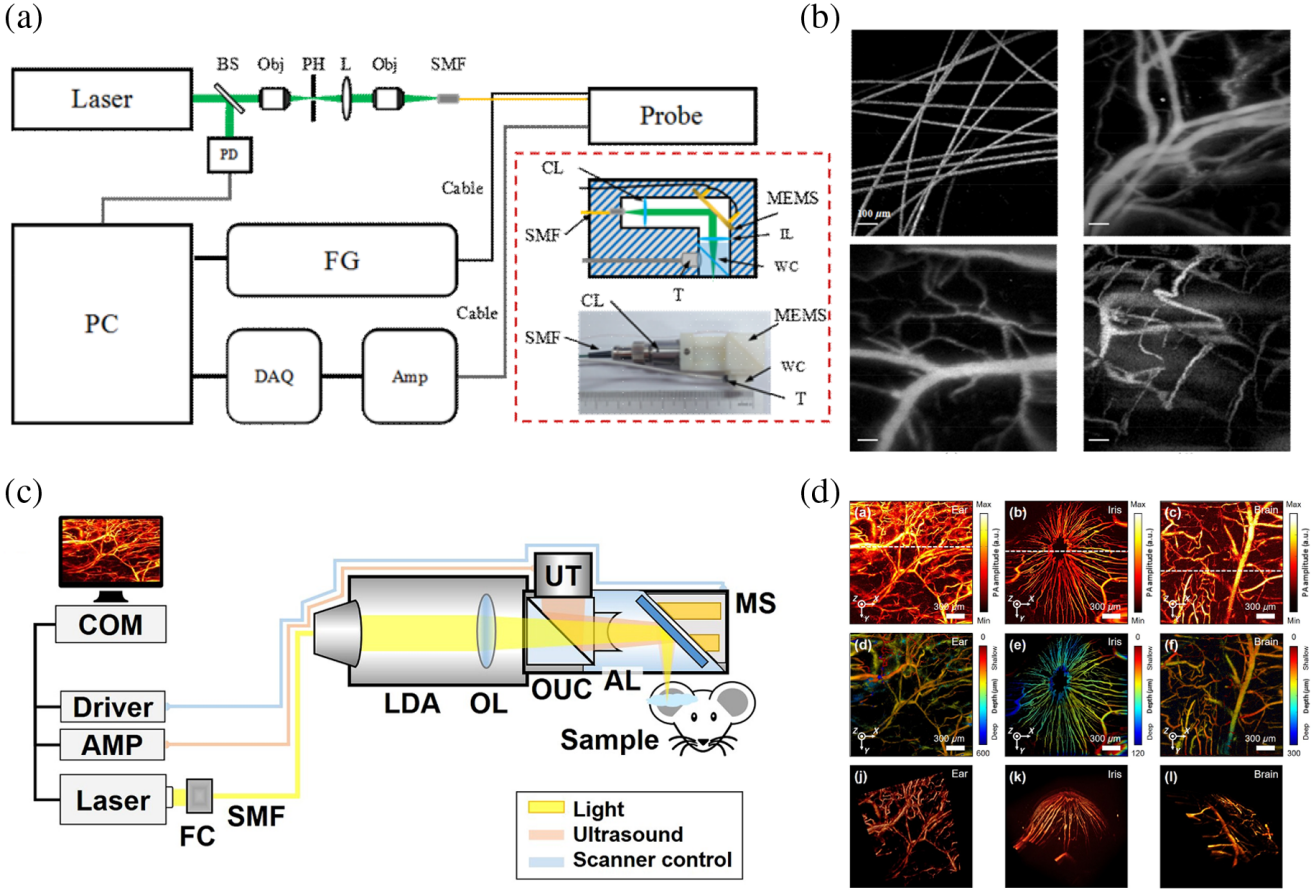
MEMS-based PAM. (a) A schematic of a miniaturized 2D MEMS-based reflection-mode PAM system. (b) PAM images of carbon fibers, *in vivo* microvasculature in mouse ear, brain, and human lip, respectively. (c) A schematic of a MEMS-based handheld PAM probe system. (d) PA MAP images, depth map images, and 3D PA volumetric images of a mouse ear, iris, and brain, respectively. Reproduced with permission from Refs. 61 and 62.

Chen et al.^59^ developed a miniaturized probe head of a PAM system using a MEMS mirror. The MEMS mirror was placed after the fiber-based pulsed laser to perform 2D scanning of the laser beam with a maximum scanning area of ∼2.3  mm×3.5  mm. A microring detector was used to detect the PA signals in a transmission mode with a lateral resolution of 17.5  μm and an axial resolution of 20  μm. In addition, they further incorporated the confocal fluorescence microscopy imaging modality into the miniaturized system without adding bulk to the probe, demonstrating the capability of the dual-modality miniaturized system for both microvasculature and individual cell imaging.^60^ However, the transmission-mode design limits its potential in *in vivo* applications. Qi et al.^61^ utilized a 2D MEMS scanning mirror to develop a miniaturized reflection-mode PAM system, shown in Fig. 4(a). They integrated optical components, a MEMS scanning mirror, and a flat UT into a cubic probe with a volume of 60  mm×30  mm×20  mm and a weight of 40 g. Figure 4(b) shows PAM imaging of carbon fibers of the *in vivo* microvasculature in mouse ear, brain, and human lip, respectively, with a lateral resolution of 10.4  μm and an active imaging area of 0.9  mm×0.9  mm. Subsequently, Chen et al.^63^ made further advancements by enhancing the resolution to 3.8  μm and expanding the effective imaging area to 2  mm×2  mm. They achieved these improvements while simultaneously reducing the probe size to merely 22  mm×30  mm×13  mm and reducing its weight to 20 g. *In vivo* experiments conducted on the internal organs of a rat abdominal cavity and entire oral cavities of volunteers demonstrate the remarkable optimization of both the performance and physical characteristics of the system.

In the aforementioned MEMS-based PAM systems, the scanning of only optical beams by the MEMS scanner limits the imaging FOV due to the efficient aperture of the UT. Lin et al.^64^ presented a handheld PAM probe, by which both optical and acoustic beams were scanned confocally through a 2D water-immersible MEMS scanner with an imaging rate of 2 Hz over a 2.5  mm×2.0  mm×0.5  mm volume. Longitudinal experiments were also conducted to verify the system’s capability for monitoring the hemoglobin concentration in blood vessels. Nevertheless, there is still room for further optimization in reducing the probe size, which currently measures at 80  mm×115  mm×150  mm. Figure 4(c) shows another MEMS-based handheld PAM probe with a diameter of 17 mm and a weight of 162 g.^62^ An opto-ultrasound combiner and an aluminum mirror on the MEMS scanner were used to facilitate confocal and co-axial alignment of both light and ultrasound, achieving a high SNR (∼40  dB for the 6  μm diameter carbon fiber) and a high lateral resolution (∼12  μm). Due to the enhanced imaging performance, this system can screen microvasculature in a live animal without the need for data averaging. The mouse ear, iris, and brain were imaged by the handheld PAM system for *in vivo* demonstration. Figure 4(d) shows the MAP images, depth map images, and 3D PA volumetric images of a mouse ear, iris, and brain, respectively, providing distinct clarity in visualizing artery and vein pairs, capillaries, and brain cortical vessels. The imaging FOV of 2  mm×2  mm is sufficient to cover the entire iris in a single scan for the visualization of the microvascular morphology. For clinical studies, the handheld PAM system was used to image a mole on a human’s finger, providing valuable diagnostic information on the depth and boundary of moles. Subsequently, Qin et al.^65^ developed a handheld dual-modality imaging microscope, which integrates MEMS-based OR-PAM and OCT, to provide complementary imaging contrasts. It has a remarkably lightweight design of 35.4 g and a compact volume of 65  mm×30  mm×18  mm, providing an effective imaging area of 2  mm×2  mm. The dual-modality microscope achieves lateral resolutions of 3.7 (PAM) and 5.6  μm (OCT), along with axial resolutions of 120 (PAM) and 7.3  μm (OCT). The animal and human oral imaging experiments have successfully demonstrated its clinical feasibility.

## 5 Perspective Applications

Significant progress has been made in the development of PAM systems based on low-cost and compact light sources as well as miniaturized and handheld designs. These advancements in PAM systems have opened exciting possibilities for a wide range of clinical applications, from benchtop to bedside. Promising perspective applications of PAM have emerged due to the improved accessibility and affordability as well as the enhanced portability and versatility, giving them more potential to be translated to various areas of medical research, diagnosis, and clinical practice.

Conventional PAM systems have low feasibility to image narrow areas such as the oral cavity, throat, cervix, and abdominal viscera. Taking advantage of the above low-cost and compact designs, a new generation of PAM systems has emerged and overcome this challenge, enabling effective imaging in previously hard-to-reach anatomical regions. Zhang et al.^66^ developed a miniaturized PAM probe and integrated it into an imaging pen with a 12 mm diameter of the distal head, which can be handheld and perform dual-view (forward and lateral) detection *in vivo* with a 2.4 mm diameter imaging area. To be adapted to different axial depths of blood vessels in different regions, an axial adjuster with a doublet lens has been designed, enabling fine adjustment of the axial position of the light focus to ensure optimal imaging quality. In addition, a 2D MEMS scanner combined with an SMF was employed for excitation light scanning in two modes. In clinical practices, the PAM pen first works in the high-speed imaging mode (4 frames per second) to find the areas of interest, followed by a high-resolution imaging mode (a lateral resolution of 18.2  μm) to acquire detailed images for more accurate diagnosis. Lu et al.^67^ also proposed a MEMS-based handheld PA laparoscopy system for narrow-area PA imaging. In addition, they combined an adaptive resampling method with the system for nonlinear distortion correction to further optimize the performance of the system.

Conventional PAM systems have been widely explored to study the cardiovascular and neurological diseases of anesthetized animals. There is a challenge for conventional PAM systems to investigate the morphological and functional information of vasculature in freely moving animals. However, due to the development of low-cost, miniaturized, and handheld PAM systems, various attempts have been made to achieve a low-cost, wearable, and robust PAM system for preclinical cardiovascular or neurological studies of freely behaving animals as well as some clinical long-period monitoring applications. For instance, Chen et al.^68^ reported a wearable PAM system, which integrates all optical, acoustic, and MEMS mechanical scanning components into a small volume imaging probe with a weight of 8 g, to monitor the cerebral hemodynamics of awake and freely moving rodents. A high-resolution (∼2.25  μm) image with a 1.2  mm×1.2  mm FOV can be acquired in 10 s. Longitudinal experiments have demonstrated the ability of the wearable PAM system to monitor cerebral hemodynamic responses to ischemia and reperfusion. Further improvement has been made to integrate PAM and electroencephalograph for comprehensive observation of both vascular and neural activities.^69^ Another fiber-coupled LD-based compact PAM system has also been developed to meet wearable applications.^70^ Instead of an expensive and bulky pumped laser, a fiber-coupled PLD was used for excitation. Furthermore, the integration of a miniaturized ring transducer (∼2.5  mm diameter) and compact translational stages also contributes to the reduction in size and weight of the wearable PAM system.

## 6 Summary

Miniaturized PAM systems have the potential to revolutionize biomedical imaging by providing high-resolution, noninvasive, and real-time imaging in a compact and affordable form. In this review, we have discussed recent efforts in developing cost-efficient and compact PAM systems. We highlight the significant progress made in developing low-cost and compact light sources, such as PLDs and CWLDs, as alternatives to expensive pumped lasers, listed in Table 1. In addition, we explore some miniaturization efforts in PAM system design with laser delivery and scanning protocols. The summary of the miniaturization design of PAM systems categorized based on the scanning protocol is presented in Table 2.

**Table 1:** Summary of PAM systems with low-cost and compact excitation sources.

Sources	Ref.	Remark	Targets	Lateral resolution	Image quality	Advantage	Disadvantage
PLDs	30	NIR	Knotted and helical blood vessels phantom	500 μm	20.6 dB (128 averaging)	Deep penetration depth; simple excitation technique	Low SNR; challenging for low-loss focusing
	32,33	NIR	Dead ant	1.5 μm	11 dB (128 averaging)		
	37	NIR	Polyethylene tubes filled with blood; *in vivo* mouse ear	7 μm	12.7 dB (128 averaging)		
	34	NIR	*Ex vivo* mouse ear and porcine ovarian	40 μm	22 to 25 dB (128 averaging)		
	39	NIR; laser scanning	*Ex vivo* mouse ear and porcine ovarian	21 μm	12 to 18 dB		
	40	NIR; square-core MMF	*Ex vivo* rabbit ear	10.3 μm	25 dB (1200 averaging)		
	41	405 nm	Carbon fibers; *ex vivo* mouse back	0.95 μm	—	High resolution	Low SNR; low pulse energy
	36	450 nm; reflective objective	*In vivo* mouse ear	10 μm	14 dB (64 averaging)	Long working distance	Low SNR
CWLDs	43	405 nm; CW mode with intensity modulation	Red blood cells in a blood smear	0.75 μm	—	High resolution	Complicated data processing; limited penetration depth
	46	450 nm; pulsed mode by overdriving	*In vivo* mouse ear and human forearm	110 μm	15 to 20 dB (500 averaging)	Simple excitation technique	Low resolution and SNR; limited penetration depth
	44	450 nm; pulsed mode by overdriving	Leaf phantom; *in vivo* mouse ear	4.8 μm	31 to 42 dB	Simple excitation technique; high resolution and SNR	Limited penetration depth
	45	450 nm and 532 nm; pulsed mode by overdriving	*In vivo* mouse ear with ${\mathrm{sO}}_{2}$ measurement	6 μm	28.3 to 31.6 dB		

**Table 2:** Summary of PAM systems with miniaturized designs.

Scanner	Ref.	Portability	Imaging range	Lateral resolution	Image speed	Samples
GVS with raster scan	50	Handheld	400 μm×400 μm	7 μm	400 Hz/B-scan	Carbon fibers; *in vivo* mouse ear
		40 mm × 60 mm				
		<500 g				
	52	Tabletop	2 mm × 2 mm	8.9 μm	25 Hz/B-scan	Leaf phantom; *in vivo* rooster’s wattle; *in vivo* human lip and wrist
	53	Handheld	14.5 mm × 9 mm	11.5 μm	32 s/volumetric scan	Carbon fibers; leaf phantom; *in vivo* mouse ear, iris, and brain
	54	Handheld	1.7 mm × 5 mm	6.2 μm	1288 Hz/B-scan	*In vivo* mouse ear, skin, intestine, stomach, kidney, liver, heart, and brain; with sO_2_ measurement
		59 mm × 30 mm × 44 mm				
		158 g				
GVS with rotatory scan	55–57	Tabletop	$8.4^{2}\;\mathrm{mm}\times \pi \;\mathrm{mm}$	10.4 μm	—	*In vivo* human lip; mouse brain; *in vivo* rabbit ear and eye
MEMS	59,60	Tabletop	2.3 mm × 3.5 mm	17.5 μm	—	*Ex vivo* canine bladder
	61	Handheld	0.9 mm × 0.9 mm	10.4 μm	8 s/volumetric scan	*In vivo* mouse ear and brain; *in vivo* human lip
		60 mm × 30 mm × 20 mm				
		40 g				
	63	Handheld	2 mm × 2 mm	3.8 μm	—	*In vivo* rat ovary, uterus, colon, and bladder; *in vivo* human lip, pterygomandibular fold, tongue, and gum
		22 mm × 30 mm × 13 mm				
		20 g				
	64	Handheld	2.5 mm × 2 mm	5 μm	0.5 s/volumetric scan	*In vivo* mouse ear; *in vivo* human skin
		80 mm × 115 mm × 150 mm				
	62	Handheld	2 mm × 2 mm	12 μm	35 Hz/B-scan	*In vivo* mouse ear, iris, and brain; *in vivo* human mole
		17 mm in diameter				
		162 g				
	65	Handheld	2 mm × 2 mm	3.7 μm	—	Carbon fibers; *in vivo* mouse ear; *in vivo* human lip and tongue
		65 mm × 30 mm × 18 mm				
		35.4 g				
	66	Imaging pen	$2.4^{2}\;\mathrm{mm}\times \pi \;\mathrm{mm}$	8.2 μm	—	Human oral cavity
		12 mm in diameter				
	67	Imaging pen	1.2 mm × 0.8 mm	12.7 μm	20 Hz/B-scan	*In vivo* rat intestines surface
	68	Wearable	1.2 mm × 1.2 mm	2.25 μm	10 s/volumetric scan	*In vivo* mouse brain
		8 g				
Miniaturized stages	70	Wearable	3 mm × 3 mm	10 μm	60 s/volumetric scan	Pencil lead

However, several challenges remain to be overcome for the cost-efficient and miniaturized PAM: (1) the LD output beam suffers from significant divergence, necessitating the use of additional optics or fibers to achieve tight focusing for high-resolution imaging. (2) The output energy of LDs is less than that of a typical pumped laser, which may degrade the SNR and imaging depth of the system. (3) Although scanning devices are used to increase the imaging speed and minimize the size of PAM systems, there is still a tradeoff among imaging FOV, resolution, and speed. Therefore, there is a strong desire for further advancements in technical development to enhance the performance of the low-cost and compact PAM system as an imaging tool in the biomedical field.

In summary, the findings presented in this review underscore the potential of cost-effective and miniaturized PAM systems to offer new possibilities for preclinical and long-term monitoring applications across diverse fields. In addition, the current miniaturized PAM systems predominantly use traditional pumped lasers, but there is potential for further development of LD-based miniaturized PAM systems. High-repetition-rate LDs combined with fast scanning strategies (e.g., GVS and MEMS) can enable large FOV and high-speed imaging, making LD-based miniaturized PAM systems a promising direction. These advancements will further facilitate the commercialization of PAM.

## Code, Data, and Materials Availability

All data in support of the findings of this paper are available within the article.
